# *TREM2*, Driving the Microglial Polarization, Has a *TLR4* Sensitivity Profile After Subarachnoid Hemorrhage

**DOI:** 10.3389/fcell.2021.693342

**Published:** 2021-08-11

**Authors:** Yangchun Hu, Chao Li, Xiaojian Wang, Weiwei Chen, Yu Qian, Xingliang Dai

**Affiliations:** Department of Neurosurgery, The First Affiliated Hospital of Anhui Medical University, Hefei, China

**Keywords:** subarachnoid hemorrhage, neuroinflammation, microglia, *TREM2*, *TLR4*

## Abstract

Increasing evidence suggests that triggering receptor expressed on myeloid cells 2 (*TREM2*) is implicated in the pathophysiology of neuroinflammation. The aim here was to investigate the neuroprotective role of *TREM2* and its regulatory mechanism after subarachnoid hemorrhage (SAH). *TREM2* siRNA was administered to measure the detrimental role of *TREM2* in mediating microglial polarization *in vivo* and *in vitro* after experimental SAH. The relationship between Toll-like receptor 4 (*TLR4*) signaling and *TREM2* was further explored. The soluble *TREM2* from the cerebrospinal fluid (CSF) of patients with SAH was detected. The results showed that *TREM2* mainly located in the microglia and presented a markedly delayed elevation after SAH. *TREM2* knockdown triggered increased pro-inflammatory productions, aggravated microglial activities, and further exacerbated neurological dysfunction after SAH. Significantly, *TLR4* knockout increased the expression of *TREM2*, accompanied by ameliorated neuroinflammation and improved neurological function. Corresponding to different clinical Hunt–Hess grades, obviously enhanced accumulation of soluble *TREM2* was detected in the CSF of patients with SAH. *TREM2* played a pivotal role in mediating microglial polarization after SAH, and the neuroprotective effect of *TREM2* might be potentially suppressed by the hyperactive *TLR4* in the early phase of SAH. Pharmacological targeting of *TREM2* may be a promising strategy for SAH therapy.

## Introduction

Substantial existing evidence suggests that the activation of microglia after subarachnoid hemorrhage (SAH) insult is spatiotemporally controlled, resulting in a biphasic effect (Akamatsu et al., 2019). Microglia is demonstrated to exacerbate neuroinflammation in the acute phase of SAH; nevertheless, in the recovery phase, it can also alleviate the excessive neuroinflammation and facilitate tissue repair. The switch between pro-inflammatory and anti-inflammatory may depend on the selective activation of the key receptors on microglial cell membrane (García et al., 2019).

Toll-like receptor 4 (*TLR4*) and triggering receptor expressed on myeloid cells 2 (*TREM2*) are considered the main receptors to induce a pro-inflammatory and anti-inflammatory phenotype, respectively (Zhou J. et al., 2019; Lyu et al., 2020). After SAH onset, the activated *TLR4* can immediately recruit the special adaptor elements to induce downstream transduction signals, such as nuclear factor-κB (NF-κB) and mitogen-activated protein kinases (MAPKs) (Akamatsu et al., 2019). In order to trigger an exaggerated pro-inflammatory response, *TLR4* cannot only repress nuclear receptor-dependent anti-inflammatory gene expression in the activation phase of the inflammation progress (Owens et al., 2017) but also aggravate the accumulation of matrix metalloproteinases (*MMPs*) to further inhibit the endogenous anti-inflammatory effect (Gunner et al., 2019).

*TREM2*, a pivotal endogenous anti-inflammatory receptor on microglial membrane, can be activated by the pathogen-associated molecular patterns (*PAMPs*) and the damage-associated molecular patterns (DAMPs) (Painter et al., 2015; Konishi and Kiyama, 2018). After the activation, *TREM2* couples with adaptor proteins, resulting in altered immune responses of microglia, such as promoting cell survival, facilitating phagocytosis, and suppressing the inflammatory response (Wu et al., 2017; Konishi and Kiyama, 2018). At the transcription level, *TREM2* can be directly regulated by nuclear factors, which have the ability to drive the resolution of inflammation (Lefterov et al., 2015). Additionally, the ectodomain of full-length *TREM2* receptor can be cleaved into a soluble form (s*TREM2*) by a disintegrin and metalloproteinase (ADAM) and released into the extracellular space (Kleinberger et al., 2014). Elevated levels of s*TREM2* have been detected in the plasma and (or) cerebrospinal fluid (CSF) of patients with neurological diseases, such as Alzheimer’s disease (AD) and ischemia (Suárez-Calvet et al., 2016; Kwon et al., 2020).

It has been demonstrated that *TREM2* could be effectively activated by the DAMPs, such as high-mobility group box protein 1 (*HMGB1*), galectin-3, *ApoE*, which also could trigger *TLR4* and its downstream pro-inflammatory pathways (García et al., 2019; Fitz et al., 2021). It is worth noting that under the same stimuli, the nerve damage caused by the pro-inflammatory response far outweighs the protective effect from the endogenic anti-inflammation effect in the early phase of acute brain injury, resulting in irreparable neurological damage. Thus, we speculated that there might be a mutual regulation relationship between the two mechanisms of pro-inflammatory and anti-inflammatory, which ultimately affected the outcome of nerve injury and repair at different stages. Strikingly, it has also been reported that the anti-inflammation effect of *TREM2* in microglia could be partly suppressed by lipopolysaccharide (LPS) administration (Liu et al., 2020). This possibility hinted us to investigate the dynamic activation of microglial *TREM2* and its regulatory mechanism after SAH. In this present study, we hypothesized that in the early stage of SAH, (1) *TREM2* could ameliorate the SAH-induced neurological deficits by suppressing neuroinflammation, facilitating phagocytosis, and promoting lipid metabolism; (2) *TLR4* signaling could drive the ectodomain shedding of *TREM2* on the microglial membrane; and (3) *TLR4* signaling could suppress the expression of *TREM2*.

## Materials and Methods

### Animal Preparation

All animal experiments were conducted in compliance with the protocols of the Institutional Animal Care and Use Committee at Anhui Medical University, which were consistent with the National Institutes of Health Guidelines for the Care and Use of Laboratory Animals in Neuroscience Research and ARRIVE guidelines (Animal Research: Reporting of *In Vivo* Experiments). Wild-type (WT) C57BL/6 mice (8–10 weeks old) were purchased from the Animal Center of Anhui Medical University. The *TLR4* gene knockout (KO) mice originally were generated by Nanjing Biomedical Research Institute of Nanjing University. Before the collection of CSF samples, all patients signed informed consents, and the study regarding human was authorized by the local ethics committee in accordance with the Declaration of Helsinki.

### Subarachnoid Hemorrhage Model *in vivo*

The mice were well-performed SAH models *in vivo* by prechiasmatic injection of non-heparinized arterial blood (Schallner et al., 2015). The head of the mouse was fixed to a stereotactic device following deep anesthesia. The skin was sterilized to expose the bregma. After a hole was drilled into the skull (1-mm diameter, 4.5 mm in front of the bregma), non-heparinized arterial blood (60 μl) from the left ventricle of the donor mouse was slowly drawn and injected into the subarachnoid space, at a 40 angle along the previous borehole. To avoid backflow, bone wax was used to close the hole. The mice in the sham group were injected with 60 μl of saline, instead of arterial blood.

### Tissue Preparation

After deep anesthesia, mice were perfused with 50 ml phosphate-buffered saline (PBS) (1×, 4°C). The inferior basal temporal lobe adjacent to the clotted blood was harvested and immediately stored in −80°C liquid nitrogen for extraction of protein and RNA. For immunohistochemistry analysis, the brains were perfused with 50 ml PBS (1×, 4°C) and fixed in 4% paraformaldehyde overnight at 4°C. After transferring to 15 and 30% sucrose (4°C), the mouse brains were embedded for cryostat sectioning.

### Primary Cell Culture and Subarachnoid Hemorrhage Model *in vitro*

#### Primary Microglial Cell Culture

Primary microglial cells were cultured from neonatal mice at P1–P2 days (Zhong et al., 2019). Under the microscope, the leptomeninges and vascular tissues on the brain surface were dissected with microsurgical instruments. After cutting with a micro-scissor, the tissues were digested, suspended, and centrifuged. Then, the cells were plated onto flasks and cultured in Dulbecco’s modified Eagle’s medium (DMEM) supplemented with penicillin-streptomycin and fetal bovine serum (FBS) in a humidified atmosphere with 5% CO_2_ at 37°C. The medium, containing macrophage colony-stimulating factor (M-CSF, 25 ng/ml) and FBS, was changed on the third day. Then, the medium was changed every 2 days. The cells were harvested by shaking (200 rpm, 30 min) after 10–12 days in culture.

#### Primary Neuron Culture

Primary neurons were cultured from fetal mice at E16–18 days (Zhou X. et al., 2019). In brief, after digestion and centrifugation, the cell pellets were resuspended in DMEM containing FBS and penicillin-streptomycin. After 2 h, the culture medium was replaced with Neurobasal medium containing GlutaMax-I, B27 supplement, and penicillin-streptomycin. The neurons were cultured in a humidified atmosphere with 5% CO_2_ at 37°C. Then, the medium was changed every 2 days. Primary neurons were harvested for detection until day 10.

#### Subarachnoid Hemorrhage Model of Microglia *in vitro*

To mimic the SAH model *in vitro*, OxyHb (MilliporeSigma, United States) was added to the primary microglia medium, and the final concentration reached 10 mM. After the SAH model was induced, the cells were collected at different time points according to the experimental design.

### Microglia and Neuron Coculture System

To obtain the microglia and neuron coculture system (Schallner et al., 2015), microglia were plated on the upper chamber of Transwell (pore size = 0.4 mm; Corning, United States) at a density of 1 × 10^5^/cm^2^. The neurons were seeded in the plates at a density of 3 × 10^5^/cm^2^. Two kinds of cells were cultured separately for 10 days. Then, the microglia were placed onto the neurons’ wells, immediately followed by the direct OxyHb administration on microglia in the upper chamber. The medium of the coculture system was DMEM containing FBS and penicillin-streptomycin. The coculture system was maintained in a humidified atmosphere with 5% CO_2_ at 37°C.

### Lentivirus Delivery *in vivo* and *in vitro*

In order to knockdown the *TREM2* gene, the lentivirus vector (LV) expressing *TREM2* shRNA and negative control (NC) were transfected *in vivo* and *in vitro* (Hanbio, Shanghai, China). Briefly, after deep anesthesia, the head of the mouse was fixed on the stereotactic apparatus. The LV-sh*TREM2* and LV-NC at 1 × 10^9^ Tu/ml were slowly injected into the left ventricle (bregma: –0.4 mm, lateral: 1.2 mm, depth: 2.5 mm; 3 μl; 0.5 μl/min) by a 10-μl Hamilton syringe (Zhou X. et al., 2019). Modeling was carried out on the third day after lentivirus transfection *in vivo*.

*In vitro*, the LV-sh*TREM2* and LV-NC at 1 × 10^9^ Tu/ml were transfected into the microglia at a density of 1 × 10^5^/cm^2^ according to the manufacturer’s protocol. Microglia were infected with a transfection coefficient of 20 following our preliminary experiment (multiplicity of infection = number of Tu/number of cells). After 48-h incubation, the microglia were used according the experimental design. The sequences of shRNA and NC were shown in Supplementary Table 1. The knockdown level of *TREM2* was measured by immunofluorescence and PCR analyses.

### Study Design and Drug Administration

In experiment 1, the time course of endogenous changes of *TREM2 in vivo* and *in vitro* at different time points after SAH was characterized. Here, 108 mice were randomized into six groups: Sham group (*n* = 18) and five SAH groups (6 h, 1, 3, 7, and 10 days, *n* = 18/group). The primary microglial cells were randomized into five groups: Control group and four SAH groups (6, 12 h, 1, and 2 days).

In experiment 2, the detrimental role of *TREM2* in mediating microglia polarization *in vitro* after SAH was evaluated. The primary microglial cells were randomized into three groups: Control group, SAH+LV-NC group, and SAH+LV-sh*TREM2* group.

In experiment 3, the underlying regulating mechanisms between *TLR4* and *TREM2 in vitro* after SAH were investigated. The primary microglia were randomized into the following groups: (1) control group, SAH+ vehicle group, and SAH+GI254023X; (2) control group, SAH+vehicle group, and SAH+MIP group; (3) control group, WT group, and *TLR4*-KO group. Inhibitors used were ADAM10-specific inhibitor GI254023X (5 μM, Sigma) and MyD88 inhibitory peptide (MIP; 100 μM, R&D Systems) (Kleinberger et al., 2014; Yang et al., 2018).

In experiment 4, the detrimental role of *TREM2* in modulating microglial polarization *in vivo* after SAH was evaluated. Here, 72 mice were randomized into three groups: Sham group, SAH+LV-NC group, and SAH+LV-sh*TREM2* group (*n* = 24/group).

In experiment 5, the potential mechanisms of *TLR4*/*TREM2 in vivo* after SAH were investigated. Here, 162 mice were randomized into the following groups: (1) sham group, SAH+vehicle group, and SAH+GI254023X group (*n* = 12/group); (2) sham group, SAH+vehicle group, and SAH+MIP group (*n* = 12/group); (3) sham group, SAH+WT group, and SAH+*TLR4*-KO group (*n* = 30/group). Inhibitors that were intraperitoneally used were GI254023X (25 mg/kg/day) and MIP (10 mg/kg/day). Both inhibitors were previously demonstrated to cross the blood–brain barrier (Yang et al., 2018; Gunner et al., 2019).

### Western Blot Analysis

The cells were placed into six-well culture plates at a density of 1 × 10^5^ cells/well, and inferior basal temporal lobe tissues were, respectively, lysed in radioimmunoprecipitation buffer containing protease (Beyotime, Jiangsu, China). After protein concentrations were detected by bicinchoninic acid (BCA) assay kit according to the manufacturer’s instruction, equal amounts of proteins were separated on different concentration of sodium dodecyl sulfate (SDS) polyacrylamide gels and transferred to polyvinylidene fluoride (PVDF) membranes. After blocking with skim milk, primary antibodies (shown in Supplementary Table 2) were incubated overnight at 4°C. The membranes were then incubated with appropriate secondary antibodies for the following visualization with enhanced chemiluminescence (ECL) solution. Band intensities were quantified by the ImageJ software.

### Immunofluorescence and TUNEL Staining

The primary microglial cells were seeded into 24-well culture plates at a density of 1 × 10^5^ cells/well. Before the immunofluorescence analysis, the cells were washed with PBS (1×) three times and fixed with 4% paraformaldehyde for 15 min. After washing with PBS, the cells and mouse coronal sections were, respectively, incubated with 0.2% Triton X-100 and 5% sheep serum at room temperature. The samples were stained with primary antibodies (shown in Supplementary Table 3) at 4°C overnight. After incubating the fluorophore-conjugated secondary antibodies, the samples were washed with PBST three times and hatched with 4′, 6-diamidino-2-phenylindole (DAPI) at room temperature.

The terminal deoxynucleotidyl transferase-mediated dUTP nick end-labeling (TUNEL) assay was conducted following the manufacturer’s instructions (Roche). The cells and coverslips were incubated with primary antibody against neuronal nuclei at 4°C overnight and hatched with enzyme and label solution for 45 min at 37°C. The samples were stained by DAPI for 1 min and then washed three times with PBST. The staining results were observed with a fluorescence microscope (Scope A1; Carl Zeiss, Germany).

### Golgi Staining and Nissl Staining

The Golgi staining was detected following the manufacturer’s instructions of the FD Rapid Golgi Stain kit (FD NeuroTechnologies). Briefly, the mouse brains were dissected quickly and immersed in impregnation solution at room temperature for 2 weeks in the dark. After transferring into solution C for 72 h at room temperature in the dark, the brains were sectioned at a thickness of 100 μm and stained following standard staining procedures. The spine densities in the hippocampus were detected from distinct second or third dendritic terminal branches in different groups. Dendritic spine density was shown as the number of spine/10 mm.

After hydrating in toluidine blue, tissue sections were dehydrated and mounted. Quantitatively, 10 fields in each section were randomly detected, and the mean number of intact cells was counted as the result of each section. Finally, six sections from each mouse were quantified for the average numbers.

### Real-Time PCR Analysis

Briefly, RNA samples from primary cells were placed into six-well culture plates at a density of 1 × 10^5^ cells/well, inferior basal temporal lobe tissues were isolated with TRIzol reagent (Invitrogen), and cDNA were acquired by reverse transcriptase reagent (Takara) according to the manufacturer’s protocol. Gene expressions were analyzed by real-time PCR system (Roche). Primer sequences were shown in Supplementary Table 4. After calculating the threshold cycle value of each gene, the results were shown as the ratio of the relative mRNA level of target gene to the corresponding mRNA expression of β-actin. When analyzing statistical differences, the relative ratio of the control group was set to 100%, and the mRNA values of other groups were converted into multiples compared with the control group.

### Enzyme-Linked Immunosorbent Assay Detection

According to the manufacturer’s protocol, the level of s*TREM2* in patients’ CSF was detected with human *TREM2* ELISA kit (Thermo Fisher Scientific). In addition, s*TREM2* levels in supernatant of primary microglia and in Tris-buffered saline (TBS) fractions from mouse brain were quantified by mouse *TREM2* ELISA kit (LifeSpan BioSciences).

### Cell Viability Assay

The microglial cells were cultured into 96-well plates at a density of 6 × 10^4^ cells/well. The viability of primary microglial cells in different groups was detected by the Cell Proliferation Assay (Promega) according to the manufacturer’s instruction (Zhong et al., 2017). At 12 h after SAH was induced *in vitro*, the cells were directly incubated with One Solution Reagent for 60 min at 37°C (20 μl/well). Then, the absorbance at 490 nm was recorded with a spectrophotometer.

### Phagocytosis Assays

Briefly, the microglial cells were plated in 24-well plates at a density of 8.5 × 10^4^ cells/well. At 12 h after SAH was induced *in vitro*, cells were incubated with pHrodo *Escherichia coli* bioparticles (50 mg, 1 mg/ml) for 60 min at 37°C. The *E. coli* bioparticles conjugated to a dye emitting fluorescence, which can only be detected after endocytosis and transported to the lysosome of microglia. Cytochalasin D, added 30 min before the incubation of pHrodo *E. coli* bioparticles, was used as a negative control. Cells were harvested by trypsinization, washed two times, and subsequently analyzed by a MACSQuant VYB flow cytometer (Miltenyi Biotec) (Kleinberger et al., 2014).

### Short-Term Neurological Function Evaluation

Neurological deficits were quantified at 72 h post-SAH with the Modified Garcia Score system reported by Sugawara et al. (2008). The system included six tests about sensorimotor assessment (maximum scores = 18), with scores of 0–3 for each test (Supplementary Table 5).

### Morris Water Maze Detection

The Morris water maze (MWM) test here consisted of the following two phases: A 5-day consecutive navigation test and a 1-day spatial probe test (Zhou J. et al., 2019). SAH models were induced on the sixth day, and the spatial probe test was on the seventh day. After the escape latency was recorded on the seventh day, the platform was moved. The mice were started in the opposite quadrant that contained the initial platform. They were allowed to swim for 1 min, and the duration in the platform quadrant was recorded. The escape latency of navigation test and the spatial probe test were conducted to evaluate the visual spatial and learning abilities, respectively. The time spent in the target quadrant was used to assess the spatial memory ability.

### Patients’ Material Measurement

Following standard procedures, the patients’ CSF samples (*n* = 60) were obtained with lumbar puncture or ventriculostomy on 1–3 days, 4–7 days, and 8–14 days after SAH onset and immediately frozen at –80°C until use. SAH was diagnosed according to computerized tomography and (or) digital subtraction angiography. The patients, who previously experienced central nervous system (CNS) diseases, such as brain tumor, AD, Parkinson’s disease, amyotrophic lateral sclerosis, stroke, were excluded.

### Statistical Analysis

Data were shown as mean ± SEM. Data in each group were analyzed by GraphPad Prism (version 8, GraphPad Software). Differences in multiple groups were compared by one-way ANOVA or two-way ANOVA with Bonferroni’s *post hoc* test. Student’s *t*-test was used to analyze the differences between two groups. The investigator, who identified, counted, and analyzed the results in morphological staining, was blinded to the experimental designs.

## Results

### Time Course of Endogenous Level of *TREM2* After Subarachnoid Hemorrhage

**FIGURE 1:**
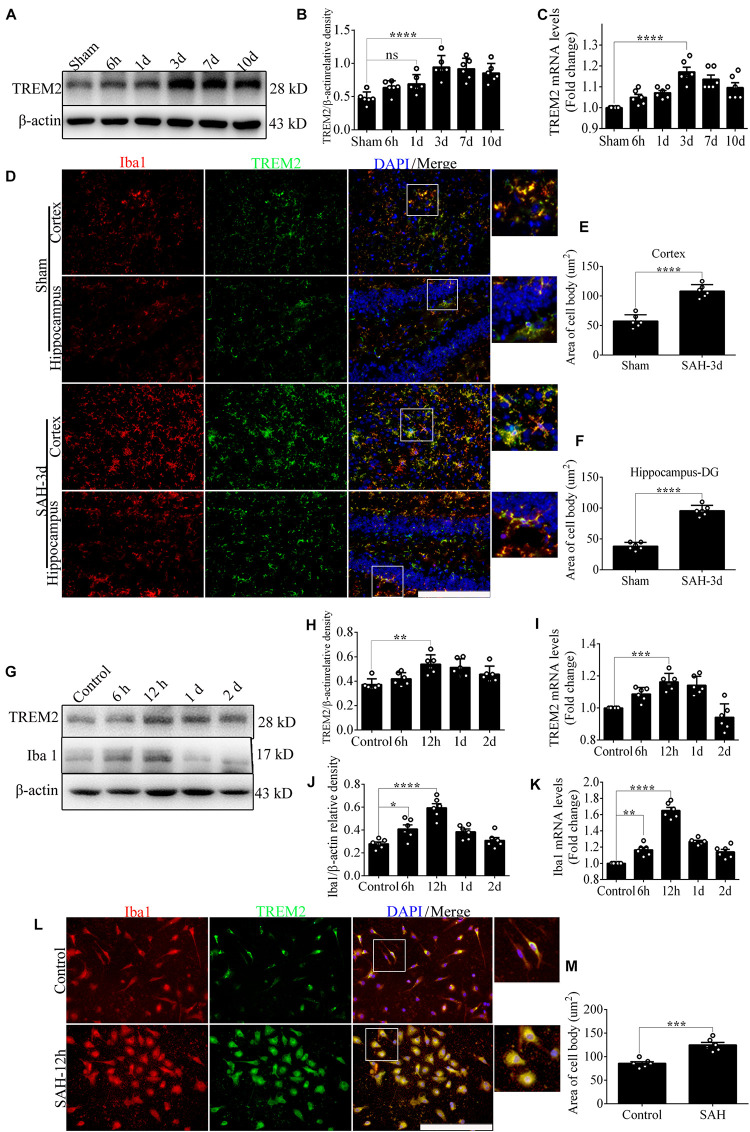
The endogenous levels of triggering receptor expressed on myeloid cells 2 (*TREM2*) *in vivo* and *in vitro* after subarachnoid hemorrhage (SAH). **(A–C)** Representative Western blot bands and quantitative analyses of *TREM2* time course *in vivo* after SAH. **(D–F)** Co-localization of *TREM2* with ionized calcium-binding adaptor molecule 1 (Iba1) was observed and quantified both in the cortex and hippocampus at 3 days after SAH. **(G–K)** Representative Western blot bands and quantitative analyses of *TREM2* and Iba1 time course *in vitro* after SAH. **(L,M)** Co-localization of *TREM2* with Iba1 was observed and quantified in primary microglia at 12 h after SAH. These data are shown as mean ± SEM (*n* = 6/group; *p<0.05, **p<0.01, ***p<0.001, ****p<0.001; scale bar = 25 μm).

*In vivo*, Western blot showed a delayed increase in *TREM2* protein level over 7 days following SAH, which peaked at 3 days and decreased gradually at 10 days. The endogenous *TREM2* mRNA level started increasing at 6 h, peaked at 3 days, and decreased at 7 days after SAH (Figures 1A–C). Ionized calcium-binding adaptor molecule 1 (Iba1), a sensitive marker of neuroinflammation, shows the existence of persistent inflammation response (Casali and Reed-Geaghan, 2021). In the sham group, co-localization revealed that *TREM2*/Iba1-positive microglia remain the resting morphology with small cell bodies and thin elongated processes. However, SAH manifestly induced microglial activation in the mice cortex and hippocampus, such as increased cell numbers, enlarged area of cell bodies, thick shorter processes (Figures 1D–F).

*In vitro*, endogenous expressions of *TREM2* and Iba1 significantly peaked at 12 h and decreased gradually at 1 day after SAH (Figures 1G–K). In the control group, immunofluorescence staining exhibited that *TREM2*/Iba1-positive microglia presented the resting morphology with small cell bodies and thin elongated processes. Obviously, SAH triggered the activation of microglia, manifested as enlarged cell bodies, thick shorter processes (Figures 1L,M).

### The Detrimental Role of *TREM2* in Mediating Microglial Polarization *in vitro* After Subarachnoid Hemorrhage

**FIGURE 2:**
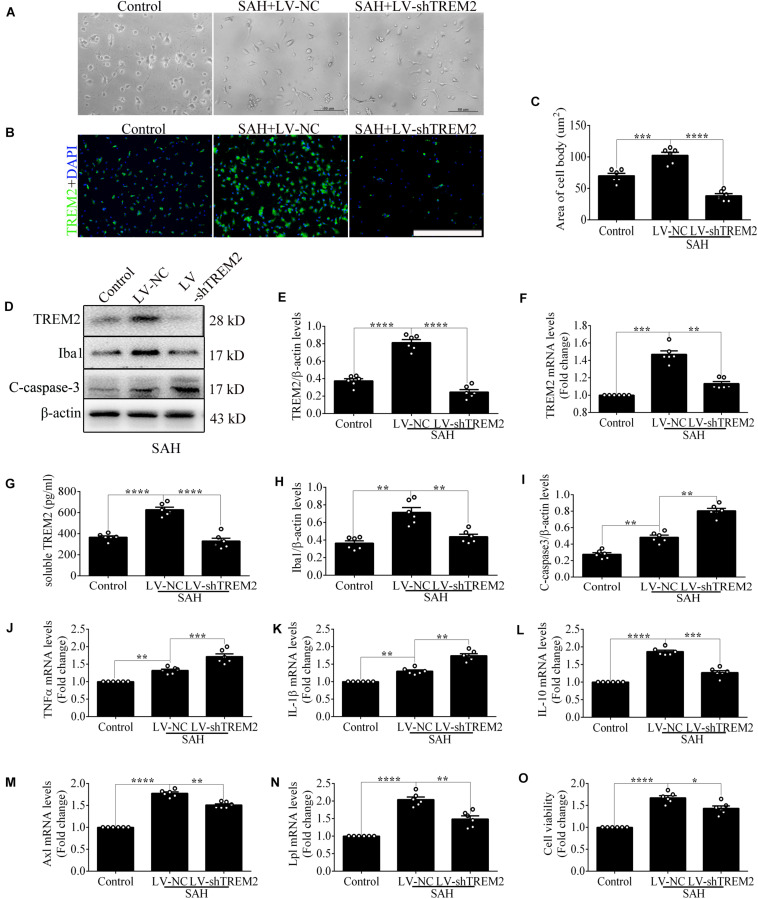
Triggering receptor expressed on myeloid cells 2 (*TREM2*) silencing modulates microglial polarization at 12 h after subarachnoid hemorrhage (SAH). **(A)** Higher-magnification images showed the morphological change of primary microglia *in vitro*. **(B,C)** Immunofluorescence staining and quantitative analysis of the knockdown efficiency of *TREM2 in vitro*. **(D–I)** Representative Western blot bands and quantification of *TREM2*, soluble *TREM2*, *TREM2* mRNA, ionized calcium-binding adaptor molecule 1(Iba1), cleaved caspase-3, and β-actin. **(J–N)** Quantitative real-time PCR detection revealed the relative mRNA levels of M2 phenotype mRNA [interleukin (IL)-10], M1 phenotype mRNA [tumor necrosis factor (TNF)α and IL-1β], phagocytic gene (Axl), and lipid metabolism-related gene (Lpl). **(O)** The changes of microglial viabilities were determined by *TREM2* knockdown. These data are shown as mean ± SEM (*p<0.05, **p<0.01, ***p<0.001, ****p<0.001; scale bar = 50 μm).

Quantitative PCR and morphological detection were used to measure the shRNA knockdown efficiency of *TREM2*. Based on the results of endogenous expression of *TREM2 in vitro*, the samples here were collected at 12 h after SAH. Compared with that in the control group, cells emerged an activated morphology with ovaloid cytoplasm and marked cellular hypertrophy in the SAH+LV-NC group. In the SAH+LV-sh*TREM2* group, few cells presented the activated state; furthermore, a considerable number of cells showed morphological atrophy (Figure 2A). After SAH, immunofluorescence staining exhibited that *TREM2*/DAPI-positive microglia obviously decreased in the LV-sh*TREM2* administration group than those in the LV-NC group (Figures 2B,C). Moreover, *TREM2* mRNA, *TREM2* protein, soluble *TREM2*, and Iba1 protein showed obviously reduced levels in SAH+LV-sh*TREM2* group than that of SAH+LV-NC group (Figures 2D–H). Moreover, the protein level of cleaved caspase-3 was markedly increased in the SAH+LV-sh*TREM2* group (Figure 2I). These results might support the possibility that the reduced level of *TREM2* exacerbates microglial survival after SAH.

Consistently, transfection of LV-sh*TREM2* significantly decreased the M2 phenotype mRNA [interleukin (IL)-10] but increased M1 phenotype mRNA [tumor necrosis factor (TNF)α and IL-1β] of activated microglia after SAH in comparison with that of LV-NC transfection (Figures 2J–L). PCR detection also showed significantly lower mRNA levels of phagocytic gene (Axl) and lipid metabolism gene (Lpl) in SAH+LV-sh*TREM2* group than that of SAH+LV-NC group (Casali and Reed-Geaghan, 2021; Figures 2M,N). Compared with the control level, microglial viability was markedly enhanced in the SAH+LV-NC group. However, viability was obviously decreased by the administration of LV-sh*TREM2* in comparison with that of LV-NC presence (Figure 2O).

**FIGURE 3:**
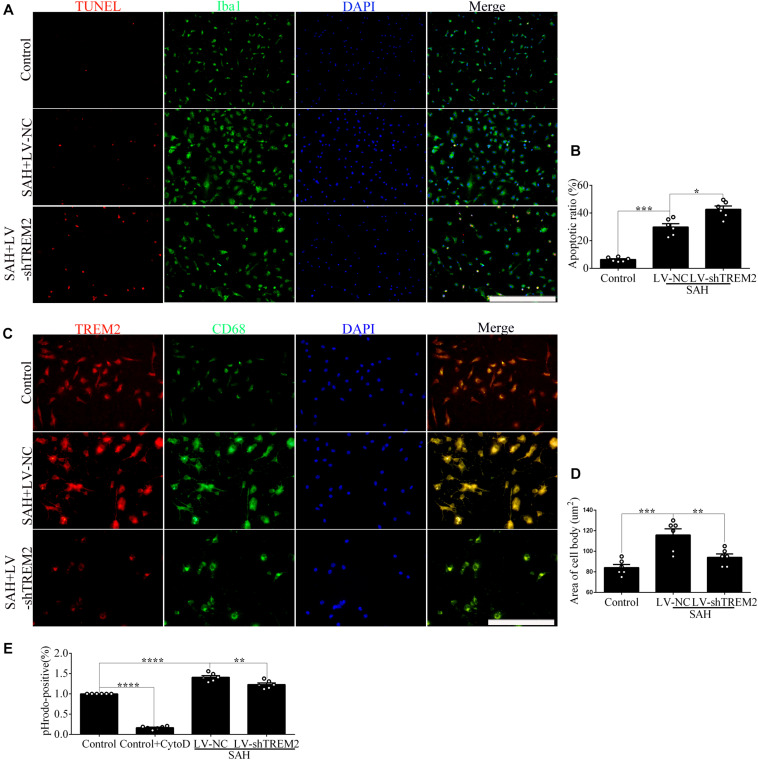
Triggering receptor expressed on myeloid cells 2 (*TREM2*) mediates the microglial survival and phagocytic capacity at 12 h following subarachnoid hemorrhage (SAH). **(A,B)** Immunofluorescence labeling and quantitative apoptotic percentage of the microglia were revealed *in vitro*. **(C,D)** Double immunofluorescence staining of *TREM2* and CD68 was detected and quantified. **(E)** The phagocytic capacity was evaluated using *Escherichia coli* conjugated to pHrodo. These data are shown as mean ± SEM (*p<0.05, **p<0.01, ***p<0.001, ****p<0.001; **A**, scale bar = 50 μm; **C**, scale bar = 25 μm).

The apoptotic ratio of microglia in the control group was low. Compared with that of the SAH+LV-NC group, the apoptotic ratio was obviously increased in the SAH+LV-sh*TREM2* group (Figures 3A,B). To evaluate the phagocytic capacity, immunofluorescence staining indirectly showed that *TREM2*-positive microglia highly presented microglial lysosome/activation marker CD68 (Linnartz-Gerlach et al., 2018; Lananna et al., 2020). Compared with that in the control level, *TREM2*/CD68 was obviously enhanced after SAH. However, the microglia in the *TREM2* silencing group reveal a lower CD68 expression, with less number of cells, than that in the SAH+LV-NC group (Figures 3C,D). Primary microglia from the SAH+LV-sh*TREM2* group demonstrated a reduced phagocytic capacity compared to SAH+LV-NC group in an assay using *E. coli* conjugated to pHrodo. Cytochalasin D, effectively inhibiting phagocytic capacity, was used as a negative control. Hence, obviously reduced amount of endocytic fluorescence was detected in the control+cytoD group in comparison with that in the control group (Figure 3E).

### Microglial *TREM2* Regulation Had a *TLR4* Sensitivity Profile *in vitro* After Subarachnoid Hemorrhage

**FIGURE 4:**
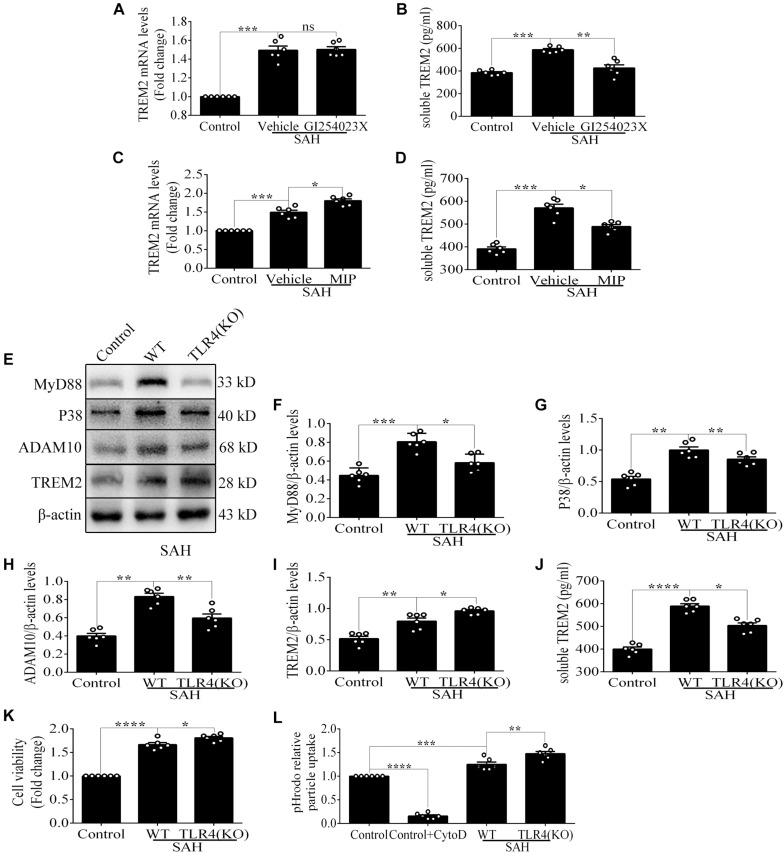
Toll-like receptor 4 (*TLR4*) pathway is involved in regulating the expression of triggering receptor expressed on myeloid cells 2 (*TREM2*) *in vitro* after subarachnoid hemorrhage (SAH). **(A–D)** Infusion with GI254023X or MyD88 inhibitory peptide (MIP) fusion protein was used to detect the regulatory effect of *TLR4* on *TREM2*. **(E–I)** The protein levels of MyD88, P38, ADAM10, *TREM2*, and β-actin were quantified by Western blotting. **(J)** ELISA detection showed the changes of soluble *TREM2*. **(K,L)** The microglial viability and phagocytic capacity were measured to assess microglial activity associated with *TREM2*. These data are shown as mean ± SEM (^ns^*p*>0.05, *p<0.05, **p <0.01, ***p<0.001, ****p<0.001).

Compared with the control level, soluble *TREM2* of microglia was obviously increased after SAH. There was a reduced level of soluble *TREM2* in the culture medium by pharmacological inhibitor of ADAM10 after SAH compared with vehicle administration. However, there were no obvious differences in the levels of *TREM2* mRNA between these two groups (Figures 4A,B), which could speculate that ADAM10 regulates *TREM2* protein by cleaving it into a secreted form after SAH. To determine whether *TREM2* expression had a *TLR4* sensitivity, we detected the changes of soluble and mRNA levels of *TREM2* after the treatment with MIP. Strikingly, we found that MIP treatment could significantly increase the mRNA level and also decrease the soluble level of *TREM2* in comparison with the vehicle treatment after SAH (Figures 4C,D). Thus, it might suggest that *TLR4*/MyD88 pathway mediates the pivotal role of *TREM2* by regulating the ectodomain shedding and gene expression after SAH. Accordingly, in contrast to WT, *TLR4* deficiency caused suppressed expressions of MyD88, P38, and ADAM10 while increasing the protein level of *TREM2* after SAH. In parallel, in comparison with that in the WT group, we observed a lower accumulation of soluble *TREM2* in the culture medium from *TLR4* deficiency primary microglia after SAH (Figures 4E–J). Compared with that in the control group, cell viability and phagocytic capacity of primary microglia were both remarkably enhanced in the WT group after SAH. Furthermore, *TLR4* deficiency reveals more high levels of cell viability and phagocytic capacity after SAH (Figures 4K,L).

**FIGURE 5:**
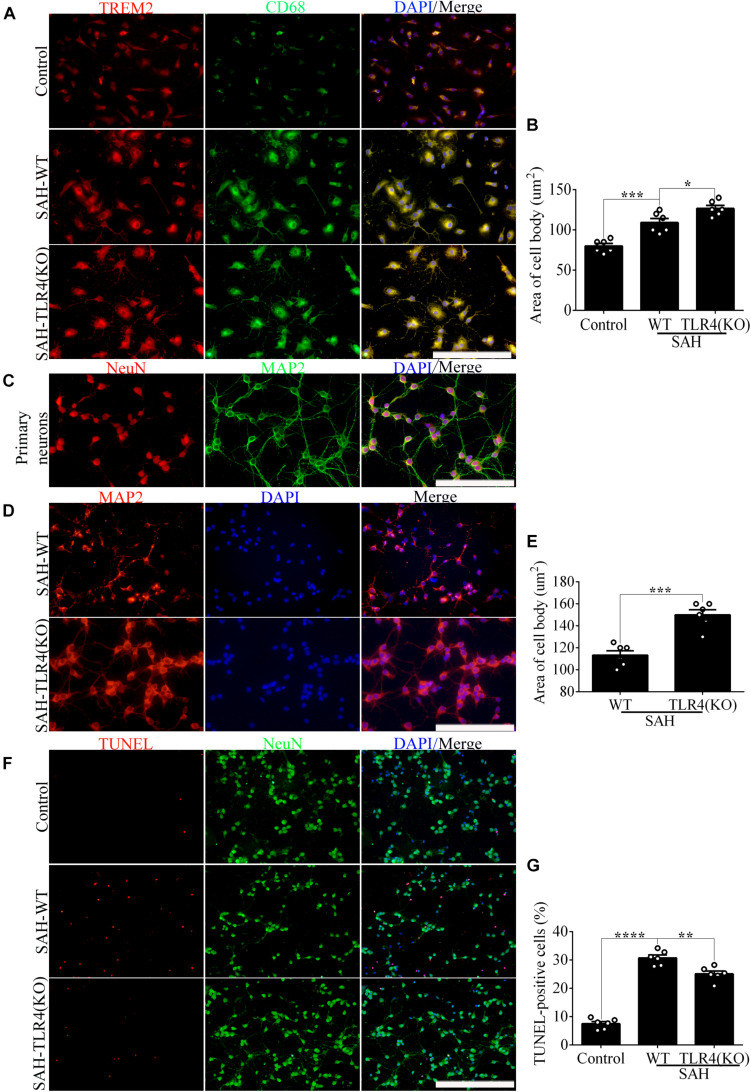
Microglial Toll-like receptor 4 (*TLR4*) deficiency ameliorates the neuronal damage in the coculture system after subarachnoid hemorrhage (SAH). **(A,B)** The double immunofluorescence staining of triggering receptor expressed on myeloid cells 2 (*TREM2*)/CD68 was detected and quantified in microglial cells. **(C)** Double immunofluorescence staining showed the cell purity of primary neurons. **(D–G)** The MAP2 staining terminal deoxynucleotidyl transferase-mediated dUTP nick end-labeling (TUNEL) labeling was quantified to reveal the regulation of microglial *TLR4*/*TREM2* on primary neurons. These data are shown as mean ± SEM (*p<0.05, **p<0.01, ***p<0.001, ****p<0.001; **A,C,D**, scale bar = 25 μm; **F**, scale bar = 50 μm).

In order to evaluate the impact of changes in microglial *TREM2* on neuronal survival after SAH, we had taken a further study in the coculture system of primary microglia and neurons. In line with the result of phagocytic capacity, immunofluorescence staining showed that *TREM2*/CD68-positive microglia in the *TLR4* deficiency group present with ovaloid cytoplasm and obvious cellular hypertrophy in comparison with the WT group after SAH (Figures 5A,B). Significantly, immunofluorescence staining showed that the cell purity of primary neurons in this study was very high (Figure 5C). In the coculture system, compared with that in the WT group, the damage of neuronal axons was obviously ameliorated in the microglial *TLR4* deficiency group after SAH (Figures 5D,E). Consistent with that, we also observed an alleviated neuronal apoptosis in the microglial *TLR4* deficiency group after SAH (Figures 5F,G). Therefore, we reasoned that microglial *TLR4* could regulate the expression of *TREM2* in the early phase of SAH and microglial *TLR4*/*TREM2* could modulate neuronal survival after SAH.

### The Pivotal Role of *TREM2* in Mediating Microglial Polarization *in vivo* After Subarachnoid Hemorrhage

**FIGURE 6:**
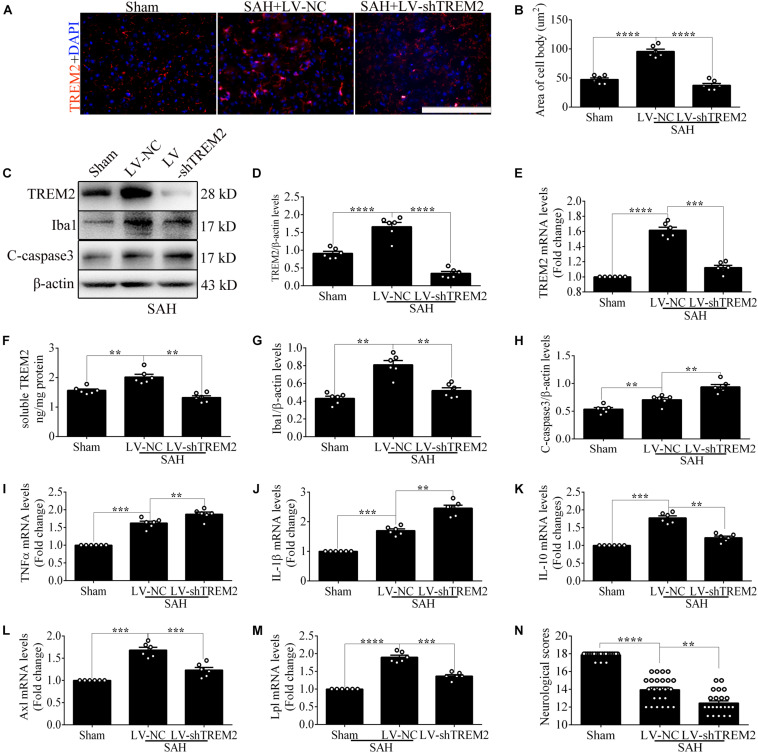
Triggering receptor expressed on myeloid cells 2 (*TREM2*) silencing regulates microglial polarization at 3 days *in vivo* after subarachnoid hemorrhage (SAH). **(A,B)** Immunofluorescence staining and quantitative analysis of the knockdown efficiency of *TREM2 in vivo*. **(C–H)** Representative Western blot bands and quantification of *TREM2*, soluble *TREM2*, *TREM2* mRNA, ionized calcium-binding adaptor molecule 1 (Iba1), cleaved caspase-3, and β-actin. **(I–M)** Quantitative PCR detection revealed the relative mRNA levels of interleukin (IL)-10, tumor necrosis factor (TNF)α, IL-1β, Axl, and Lpl. **(N)** Modified Garcia Score System showed the short-term neurological function damage. These data are shown as mean ± SEM (**p<0.01, ***p<0.001, ****p<0.001).

**FIGURE 7:**
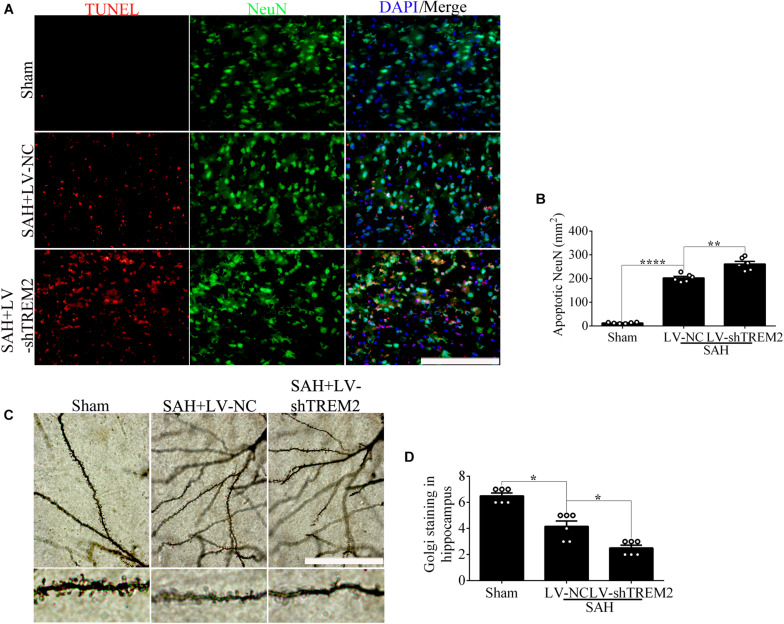
Microglial triggering receptor expressed on myeloid cells 2 (*TREM2*) silencing causes an aggravated neuronal injury at 3 days *in vivo* after subarachnoid hemorrhage (SAH). **(A,B)** Terminal deoxynucleotidyl transferase-mediated dUTP nick end-labeling (TUNEL) staining and apoptotic percentages of the neurons. **(C,D)** Golgi staining and quantitative analysis of axons. These data are shown as mean ± SEM (*p<0.05, **p<0.01, ****p<0.001; **A**, scale bar = 25 μm; **C**, scale bar = 10 μm).

Based on the results of endogenous expression of *TREM2 in vivo*, the samples here were collected at 72 h after SAH. To test the key role of *TREM2* in mediating microglial polarization, transfection of *TREM2* shRNA obviously decreased the immunofluorescence area of *TREM2 in vivo* (Figures 6A,B). In line with this finding, we similarly found the declined levels of *TREM2* protein and mRNA, accompanied by a decreased expression of Iba1. Western blot detection also revealed a manifestly higher level of cleaved caspase-3 in the LV-sh*TREM2* group than that in the LV-NC group after SAH (Figures 6C–H). Moreover, *TREM2* shRNA treatment markedly reduced the IL-10 mRNA but enhanced the TNFα and IL-1β mRNA after SAH in comparison with those in the LV-NC group (Figures 6I–K). LV-sh*TREM2* treatment showed significantly lower Axl and Lpl mRNA than those of LV-NC after SAH (Figures 6L,M). Strikingly, in comparison with the sham group, a significant impairment of behavioral function was found in the SAH+LV-NC group at 72 h. Compared with that in the LV-NC group, *TREM2* shRNA treatment showed a more severe short-term neurological function damage after SAH (Figure 6N). Consistent with that, *TREM2* shRNA injection further exacerbated the neuronal apoptosis in comparison with that by LV-NC administration after SAH (Figures 7A,B). The Golgi staining showed that the broken dendritic spine in the *TREM2* shRNA group was better preserved in the LV-NC group after SAH (Figures 7C,D). These results might indicate that *TREM2* plays a neuroprotective role in SAH mice, and that inhibition of the upregulated *TREM2* expression following SAH exacerbates neurological function.

### Microglial *TREM2* Regulation Had a *TLR4* Sensitivity Profile *in vivo* After Subarachnoid Hemorrhage

**FIGURE 8:**
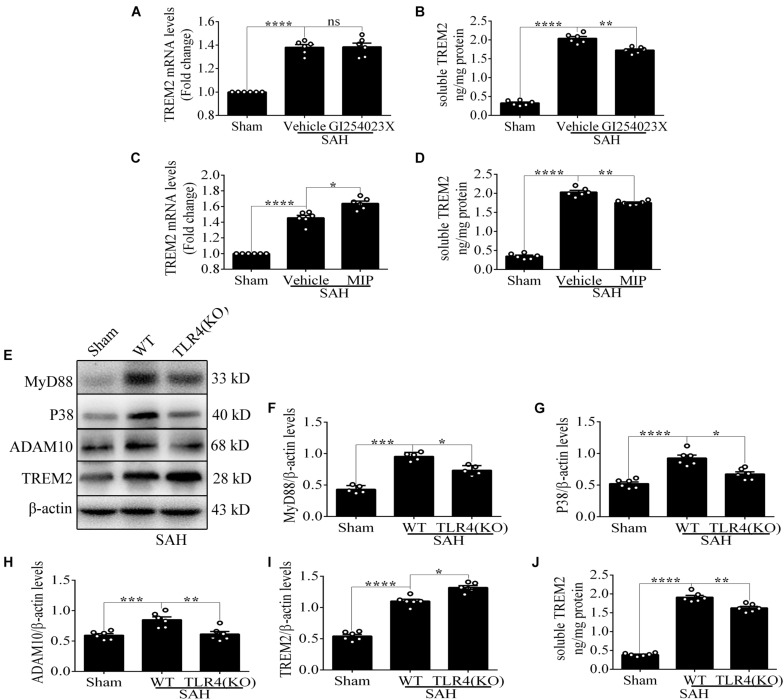
Toll-like receptor 4 (*TLR4*)/MyD88 pathway mediates the expression of triggering receptor expressed on myeloid cells 2 (*TREM2*) in mouse cortex after subarachnoid hemorrhage (SAH). **(A–D)** After the treatment with GI254023X or MyD88 inhibitory peptide (MIP) fusion protein, the relative mRNA and soluble levels of *TREM2* were detected in order to evaluate the regulatory effect of *TLR4* on *TREM2*. **(E–I)** The protein levels of MyD88, P38, ADAM10, *TREM2*, and β-actin were quantified by Western blotting. **(J)** ELISA detection showed the changes of soluble *TREM2*. These data are shown as mean ± SEM (^ns^ *p*>0.05, *p<0.05, **p<0.01, ***p<0.001, ****p<0.001).

Compared with that in the vehicle treatment group, soluble *TREM2* in the brain showed a similar decline with GI254023X treatment at 72 h after SAH. No obvious difference of *TREM2* mRNA was found between these two groups (Figures 8A,B). Additionally, MIP treatment could significantly improve the mRNA level and reduce the soluble level of *TREM2* in comparison with the vehicle treatment after SAH (Figures 8C,D). Furthermore, in contrast to the levels in WT mice, the protein levels of P38 and ADAM10 were obviously decreased in the *TLR4* deficiency group after SAH. In parallel with that, we observed an enhanced level of *TREM2* and a declined level of soluble *TREM2* in the *TLR4* deficiency group, which might speculate a suggestion that the *TLR4*/MyD88 pathway regulates the expression of *TREM2 in vivo* after SAH (Figures 8E–J).

**FIGURE 9:**
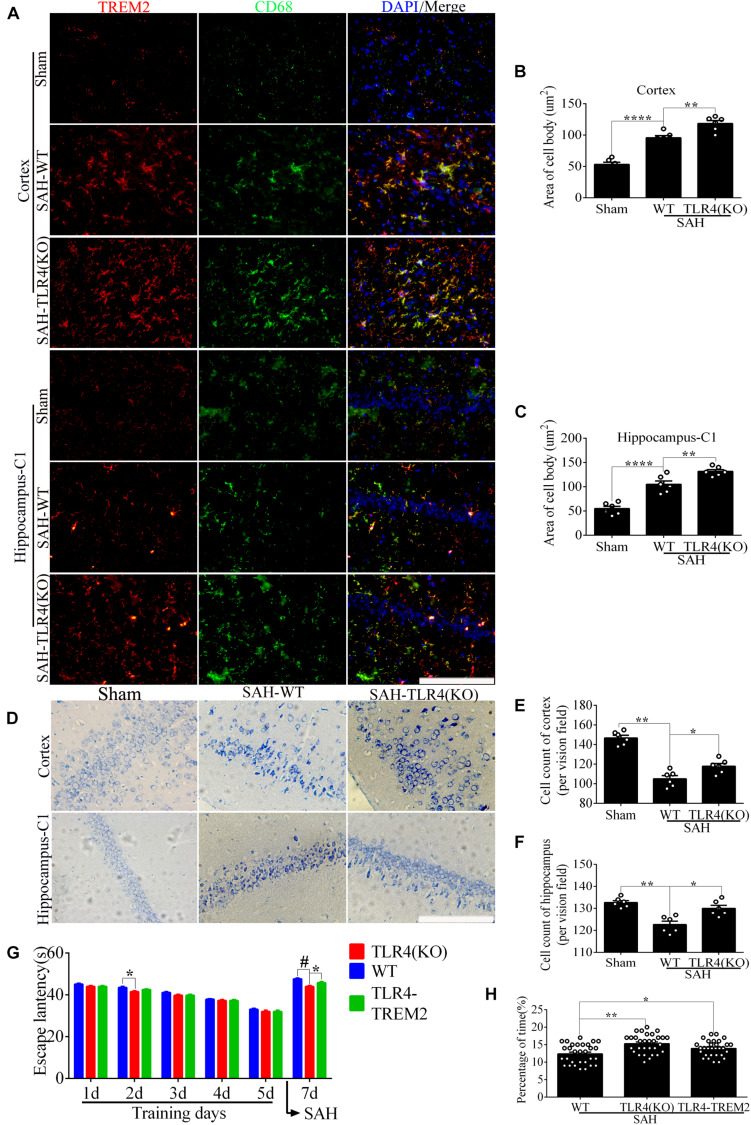
Toll-like receptor 4 (*TLR4*) deficiency ameliorates neuronal damage and improves neurological dysfunction. **(A–C)** Double immunofluorescence staining showed the triggering receptor expressed on myeloid cells 2 (*TREM2*)/CD68-positive cells in the cortex and hippocampus after subarachnoid hemorrhage (SAH). **(D–F)** Nissl staining and quantitative number of survived neurons. **(G)** Comparison of the escape latency on the navigation test on days 1, 2, 3, 4, 5, and 7. The SAH models were performed on day 6. **(H)** Comparison of the time spent in the target quadrant after removal of the platform on day 7. These data are shown as mean ± SEM (*TLR4*-*TREM2*: Knocking down the *TREM2* in the *TLR4*-KO mice; *p<0.05, **p<0.01, ***p<0.001, ***p<0.001, ****p<0.001, ^#^*p*<0.001; scale bar = 25 μm).

In the WT brain, SAH induced evident *TREM2*/CD68-positive microglial cells; however, the number of positive cells in the cortex and hippocampus was significantly increased in the *TLR4* deficiency group (Figures 9A–C). As shown (Figures 9D–F), *TLR4* deficiency significantly improved the proportion of survival neurons in comparison with the WT after SAH. In the MWM detection, we found that the latency time of KO mice was significantly reduced compared with WT mice at the second navigation test day. It indicated that KO mice had stronger adaptive ability and memory ability, which was consistent with a previous study that *TLR4* deficiency ameliorated cognition decline due to neuroinflammation in mice (Zhong et al., 2020). Following SAH model induced on day 6, the escape latency of WT mice was longer than that of the *TLR4* deficiency mice on day 7. In addition, knocking down *TREM2* in the *TLR4*-KO mice could produce a rescue of the WT phenotype after SAH (Figures 9G,H).

Taken together, these results supported the possibility that *TLR4* had a regulation on microglial *TREM2*, which effectively drove the initiation of neuronal insult and neurological dysfunction in the early phase of SAH.

### Enhanced s*TREM2* Levels in the Cerebrospinal Fluid of Patients With Subarachnoid Hemorrhage

**FIGURE 10:**
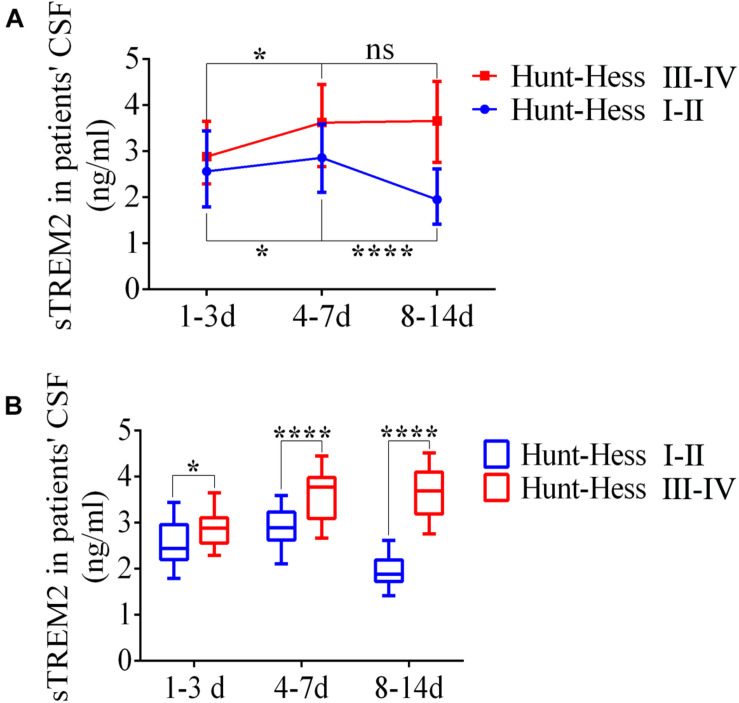
Enhanced levels of soluble triggering receptor expressed on myeloid cells 2 (*TREM2*) in the cerebrospinal fluid (CSF) of patients with subarachnoid hemorrhage (SAH). **(A,B)** ELISA-based analysis of s*TREM2* in CSF samples of SAH patients with different Hunt–Hess grades. These data are shown as mean ± SEM (^ns^*p*>0.05, *p<0.05, ****p<0.01).

The levels of soluble *TREM2* in the CSF of SAH patients were measured by ELISA according to the time period after SAH. From days 1–3 to days 4–7, the levels of s*TREM2* were significantly elevated at different Hunt–Hess grades. Respectively, compared with that on days 4–7, s*TREM2* levels of Hunt–Hess grades (I–II) patients were obviously decreased at days 8–14, while the levels present no obvious change of Hunt–Hess grades (III–IV) patients (Figure 10A). Significantly, we also found that in different time periods, patients with higher grades presented higher s*TREM2* levels (Figure 10B).

## Discussion

### The Pivotal Role of *TREM2* in Mediating Microglial Polarization After Subarachnoid Hemorrhage

Microglial cells display protective and deleterious effects at different time frames (Li and Barres, 2018; Lyu et al., 2020). In the early phase of SAH, the activation of microglial cells causes a more deleterious effect, which is considered an important therapeutic target against neuroinflammation leading to a worsened neuronal injury (Akamatsu et al., 2019). *TREM2*, a microglia-specific immunoreceptor, has been suggested to modulate versatile activity, such as suppression of pro-inflammatory cytokine, promotion of phagocytosis, enhancement of microglial proliferation and survival, and enhancement of microglial lipid metabolism (Painter et al., 2015; Keren-Shaul et al., 2017). Analogously, *TREM2* knockdown here displayed exaggerated accumulations of TNFα and IL-1β after SAH, consistent with the suggestions from some previous studies that *TREM2* had an anti-inflammatory effect (Wu et al., 2017). Additionally, we also found that *TREM2* knockdown *in vitro* obviously worsened the microglial survival after SAH, as shown by increased positive number of TUNEL staining and enhanced expression of cleaved caspase-3. These results indicated that the worsened pro-inflammatory response and deteriorated microglial survival were tightly coupled with the knockdown of *TREM2* following SAH. CD68 is a lysosome/endosome-associated membrane glycoprotein; it has been demonstrated that *TREM2*+ cells express high percentages of the phagocytic-associated molecules CD16/32 and CD68 (Manich et al., 2020). Therefore, our study well revealed that *TREM2* knockdown downregulated the microglial numbers and expression of the microglial activation marker CD68 *in vitro*, accompanied by aggravated phagocytic capability.

Microglial Lpl has been shown to mediate lipid metabolism to fuel protective phagocytic function during development, damage, and disease (Loving and Bruce, 2020; Loving et al., 2021). Due to the lack of Lpl, microglial cells are easily polarized into a pro-inflammatory state, accompanied by impaired lipid uptake and reduced fatty acid oxidation (Keren-Shaul et al., 2017). As a new role for microglial function, the expression of Lpl has a *TREM2*-dependent phase in AD (Keren-Shaul et al., 2017; Nugent et al., 2020). Consistent with that, we similarly observed a manifestly decreased level of Lpl resulting from the inhibition of *TREM2* after SAH, accompanied by exacerbated pro-inflammatory response and reduced phagocytic capability. However, the mechanism *TREM2*-associated microglial lipid metabolism regulating microglial polarization after SAH needs to be further elucidated in the future.

### *TLR4* Pathway Can Regulate the Expression of *TREM2* in the Acute Phase of Subarachnoid Hemorrhage

After SAH onset, the binding of PAMP/DAMP and *TLR4* triggers the TIR domain-containing adaptor molecules (including MyD88 and TRIF), which leads to the activation of NF-κB and MAPK pathways to accumulate pro-inflammatory cytokines. Of note, in the early phase of SAH, neuronal apoptosis was predominantly *TLR4*/MyD88-dependent and microglial-dependent (Akamatsu et al., 2019). Thus, *TLR4* is considered one of the primary factors in driving pro-inflammation following SAH. Similar to *TLR4*, *TREM2* signaling has been confirmed to be fully activated by PAMP/DAMP (Painter et al., 2015). Nonetheless, the expression of *TREM2* in our study showed a delayed increment after SAH. On the one hand, we speculated that the endogenous anti- and pro-inflammatory phenomena spatiotemporally coexist, and the endogenous anti-inflammation effort of *TREM2* might be subordinate to the pro-inflammation driven by *TLR4* in the initial stage of SAH. On the other hand, it was speculated that the initial of *TLR4* signaling might suppress the expression and (or) activity of *TREM2*, ultimately leading to an uncontrolled pro-inflammatory insult. In order to characterize the relationship, we found that MyD88 inhibitor could effectively enhance the expression of *TREM2* after SAH, including a decline of the soluble *TREM2 in vivo* and *in vitro*. This well confirmed the speculation that *TLR4* downstream generated the regulatory mechanism on *TREM2* in the initial stage of SAH, at least regulating the expression and ectodomain shedding of *TREM2*. Similarly, the *TLR4*-dependent regulation of *TREM2* in our study was consistent with the previous study, showing that LPS-induced hyperactive *TLR4* suppresses the expression of *TREM2* (Zhou J. et al., 2019). In addition, it had been reported that bexarotene, the specific small molecule ligand of retinoid X receptor (RXR), could upregulate the expression of *TREM2*, and chromatin immunoprecipitation (ChIP) assay was used to validate *TREM2* as the main select genes in response to RXR activation in BV2 cells (Lefterov et al., 2015). Meanwhile, RXR and its downstream anti-inflammatory effect were subjected to negative regulation by *TLR4* at the activation phase of inflammation in the macrophages (Oishi et al., 2017). Hence, we speculated that the SAH-induced *TLR4* pathway might drive the suppression on RXR/*TREM2* at the level of gene transcription.

Apart from neuroinflammation, *TLR4*/MyD88 pathway can effectively modulate the activation of MAPKs, which in turn leads to an accumulation of metalloprotease resulting in ectodomain shedding of membrane receptor (Ivashkiv et al., 2011; Akamatsu et al., 2019). It has been reported that membrane receptor CSF-1R could be cleaved by ADAM10, releasing soluble CSF-1R extracellular domain in the culture supernatants (Gunner et al., 2019). Consistent with that, we similarly observed an increment level of soluble *TREM2 in vivo* and *in vitro* after SAH. Especially after pharmacological inhibitor of ADAM10 treatment, a reduced level of soluble *TREM2* was detected after SAH. Therefore, we reasonably speculate that in the acute phase of SAH, *TLR4*/MyD88 pathway could be well activated, resulting in degradation of microglial *TREM2* receptor, which further suppresses the neuroprotective effect of *TREM2*.

Furthermore, s*TREM2* shows an obviously increased level in AD and stroke patients’ CSF (Suárez-Calvet et al., 2016; Kwon et al., 2020). In parallel, we observed an early increment of s*TREM2* in patients’ CSF after SAH, and patients with the higher Hunt–Hess grades presented more accumulation and longer peak time of s*TREM2* in comparison with that of patients with low grades. Thus, these might indicate that in the acute phase of SAH, the level of s*TREM2* in the CSF was positively associated with clinical severity, and s*TREM2* can be a significant biomarker to assess clinical severity and predict outcome. Additionally, the manifestly elevated s*TREM2* in patients’ CSF could further support the possibility that the initial activation of *TLR4*/MyD88 pathway could suppress the endogenous neuroprotective receptors by ectodomain shedding in the acute phase of SAH.

### Crosstalk Between Membrane Receptors *TLR4* and *TREM2* in Driving Neuroinflammation Following Subarachnoid Hemorrhage

The initial bleeding causes the release of endogenous inflammation-related ligands that may effectively activate not only the *TLR4*-dependent pro-inflammatory pathways but also the *TREM2*-dependent neuroprotective pathways (Painter et al., 2015; Akamatsu et al., 2019). In our study, the data indicated that *TLR4* pathway could effectively drive the progress of neuroinflammation in the early phase of SAH at least *via* regulating the expression and ectodomain shedding of *TREM2*. Remarkably, we also found that in the MWM detection, knocking down *TREM2* in the *TLR4*-KO mice could produce a rescue of the WT phenotype after SAH, which might indirectly indicate that the cognition decline induced by *TLR4* outweighs the endogenic neuroprotective effect driven by *TREM2* in the early phase of SAH. Meanwhile, it is plausible to deduce that activation might be initiated in a *TLR4*-dependent manner that involves downregulation of microglia checkpoints, followed by activation of a *TREM2*-dependent program (Keren-Shaul et al., 2017). In addition, it has been reported that *TREM2*, *via* blocking the extracellular signal-regulated kinase (ERK) pathway, attenuates *TLR4*-induced secretion of pro-inflammatory cytokines (Peng et al., 2013). Analogously, *TREM2* knockdown in our study triggered increased pro-inflammatory productions and aggravated microglial activities and further exacerbated neurological dysfunction after SAH. Collectively, these may well suggest a mutual regulatory mechanism between membrane receptors *TLR4* and *TREM2* in driving neuroinflammation, which may ultimately determine the switches between pro-inflammatory response and inflammatory suppression at different stages, maintaining the brain homeostasis after SAH.

**FIGURE 11:**
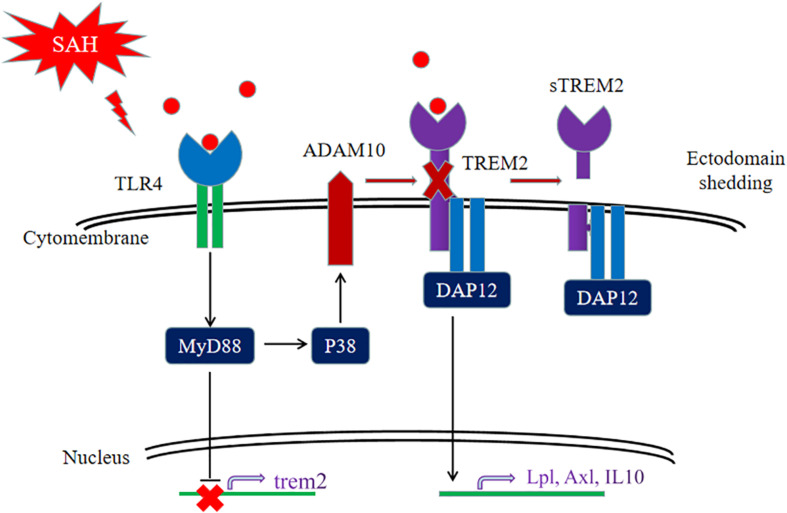
Schematic model of the interplay between triggering receptor expressed on myeloid cells 2 (*TREM2*) and Toll-like receptor 4 (*TLR4*)-induced inflammation after subarachnoid hemorrhage (SAH). The *TLR4*-induced inflammation suppresses the *TREM2* signaling pathway to exacerbate SAH-triggered inflammatory response in microglia. After SAH onset, the activated *TLR4* pathway can interfere *TREM2* by inhibiting the increment of *TREM2* expression and cleaving *TREM2* into a secreted form.

Collectively, our data firstly showed that the pivotal role of *TREM2* in mediating microglial polarization *in vitro* and *in vivo* after SAH and the neuroprotective effect of *TREM2* might be potentially suppressed by the hyperactive *TLR4* in the early phase of SAH. The SAH-induced imbalance of *TLR4*/*TREM2* effectively drove the initiation of neuroinflammation, accompanied by aggravated phagocytic capability, suppressed lipid metabolism, and exacerbated cognitive dysfunction after SAH (Figure 11). Thus, pharmacological targeting of *TREM2* may provide a new therapeutic strategy in the treatment of SAH.

## Data Availability Statement

The raw data supporting the conclusions of this article will be made available by the authors, without undue reservation.

## Ethics Statement

The studies involving human participants were reviewed and approved by the Ethics Committee at Anhui Medical University. The patients/participants provided their written informed consent to participate in this study. The animal study was reviewed and approved by the Institutional Animal Care and Use Committee at Anhui Medical University. Written informed consent was obtained from the individual(s) for the publication of any potentially identifiable images or data included in this article.

## Author Contributions

YH, CL, XW, WC, YQ, and XD participated in the experimental design, data analysis and interpretation, and manuscript preparation. YH, YQ, WC, and XD performed the experiments. CL and XW collected and analyzed the data. YH and YQ drafted the manuscript. XD proofread the language. All authors contributed to the article and approved the submitted version.

## Conflict of Interest

The authors declare that the research was conducted in the absence of any commercial or financial relationships that could be construed as a potential conflict of interest.

## Publisher’s Note

All claims expressed in this article are solely those of the authors and do not necessarily represent those of their affiliated organizations, or those of the publisher, the editors and the reviewers. Any product that may be evaluated in this article, or claim that may be made by its manufacturer, is not guaranteed or endorsed by the publisher.

## Supplementary Material

Supplementary Table 1 The sequences of shRNA.

Supplementary Table 2 Antibodies in Western blot analysis.

Supplementary Table 3 Antibodies in immunofluorescence analysis.

Supplementary Table 4 The PCR primer sequences.

Supplementary Table 5 Definitions of neurological scores.
