# Supplementary material for: TREM2, Driving the Microglial Polarization, Has a TLR4 Sensitivity Profile After Subarachnoid Hemorrhage
Source: Front Cell Dev Biol. 2021 Aug 11;9:693342. doi: 10.3389/fcell.2021.693342 (PMC8386127; doi:10.3389/fcell.2021.693342)
Supplement: Supplementary Table 1 — The sequences of shRNA. [file Data_Sheet_1.doc]

**Supplementary materials:**

**Table S1 The sequences of shRNA**

| **Gene** | **sequences of shRNA** |
| --- | --- |
| TREM2 | 5’-GAAGCGGAATGGGAGCACA-3’ |
| Negtive control (NC) | 5’-UUCUCCGAACGUGUCACGUTT-3’ |

**Table S2 Antiboties in western blot analysis**

| **Antibody** | **Company** | **Dilution** | **Catalog Number** |
| --- | --- | --- | --- |
| TREM2 | R&D Systems | 1:500 | AF1828 |
| P38 | CST | 1:1000 | 8690 |
| Iba1  cleaved-caspase3  ADAM10  MyD88 | Wako  Abcam  Abcam  CST | 1:500  1:500  1:2000  1:1000 | 016-20001  ab13847  ab124695  4283 |
| β-actin | Bioworld | 1:5000 | AP0060 |

**Table S3 Antiboties in immunofluorescent analysis**

| **Antibody** | **Company** | **Dilution** | **Catalog Number** |
| --- | --- | --- | --- |
| TREM2 | R&D Systems | 1:50 | AF1828 |
| CD68  NeuN  MAP2 | Bio-Rad  MilliporeSigma  CST | 1:50  1:300  1:300 | MCA1957  MAB377X  8707T |
| DAPI | MilliporeSigma | 1:2000 | D9542 |

**Table S4 PCR primer sequences**

| **Gene** | **Forward primer** | **Reverse primer** |
| --- | --- | --- |
| TREM2 | 5’-GCACCAACTTCAGATCCTCACT-3’ | 5’-GCAAAAGTAGCAGAAACAGAAGTC-3’ |
| TNF | 5’-CCCTCACACTCAGATCATCTTCT-3’ | 5’-GCTACGACGTGGGCTACAG-3’ |
| IL-1β | 5’-GCAACTGTTCCTGAACTCAACT-3’ | 5’-ATCTTTTGGGGTCCGTCAACT-3’ |
| IL-10 | 5’-GCTCTTACTGACTGGCATGAG-3’ | 5’-CGCAGCTCTAGGAGCATGTG-3’ |
| Lpl | 5’-CCAAGAGAAGCAGCAAGATGTA-3’ | 5’-ATCCTCAGTCCCAGAAAAGTGA-3’ |
| Axl | 5’-ATGGCCGACATTGCCAGTG-3’ | 5’-CGGTAGTAATCCCCGTTGTAGA-3’ |
| β-actin | 5’-GTCCCTCACCCTCCCAAAAG-3’ | 5’-GCTGCCTCAACACCTCAACCC-3’ |

**Table S5 Definitions of neurological scores**

| TEST | Score | | | |
| --- | --- | --- | --- | --- |
|  | 0 | 1 | 2 | 3 |
| Spontaneous  activity (in cage for 5 min) | No movement | Barely moves  position | Moves but does not approach at  least three sides of cage | Moves and  approaches at  least three sides of cage |
| Spontaneous  movements of all limbs | No movement | Slight  movement of  limbs | Moves all limbs  but slowly | Moves all limbs the same as pre-SAH |
| Movements of  forelimbs  (outstretching  while held by tail) | No  outreaching | Slight  outreaching | Outreach is  limited and less than pre-SAH | Outreach is the  same as pre-SAH |
| Climbing wall of wire cage | NA | Fails to climb | Climbs weakly | Normal climbing |
| Reaction to touch on both sides of trunk | NA | No response | Weak response | Normal response |
| Response to  vibrissae touch | NA | No response | Weak response | Normal response |

SAH, subarachnoid hemorrhage; NA, not available.
